# Hairy Cell Leukemia With Aberrant CD5 and CD23 Expression: A Case Report and a Brief Literature Review

**DOI:** 10.7759/cureus.105461

**Published:** 2026-03-18

**Authors:** Zakaria Alameddine, Rachelle Hamadi, Hagar Attia, Li Zhonghua, Robert Levy

**Affiliations:** 1 Hematology and Oncology, SUNY Downstate Health and Sciences University, Brooklyn, USA; 2 Pathology, SUNY Downstate Health Sciences University, Brooklyn, USA; 3 Pathology, Kings County Hospital Center, Brooklyn, USA; 4 Hematology and Oncology, Kings County Hospital Center, Brooklyn, USA

**Keywords:** aberrant expression, braf v600e mutation, cd5-positive hairy cell leukemia, coexpression of cd5 and cd23, hairy cell leukemia (hcl)

## Abstract

Hairy cell leukemia (HCL) is a rare indolent B-cell leukemia. It is characterized by distinctive morphologic, immunophenotypic, and molecular features. These features include the expression of typical surface markers such as CD25, CD11c, CD103, and CD123. It also includes the detection of the *BRAFV600E* mutation. Aberrant expression of CD5 and CD23 is extremely rare. We present a case of classical HCL expressing both markers, along with a brief review of the literature emphasizing the diagnostic complexities associated with such aberrancy.

## Introduction

Hairy cell leukemia (HCL) is a rare B-cell neoplasm characterized by the proliferation of small mature lymphocytes and accounts for 2% of all lymphoid leukemias [1]. The diagnosis of HCL is usually established by a combination of flow cytometry and immunohistochemistry (IHC), with the former characterized by expression of pan B cell antigens (CD19, CD20, CD22) and most of the core classical markers CD25, CD11c, CD103, and CD123 [2]. Aberrant expression is uncommon but has been described in the literature, with the majority involving the following markers: CD5+, CD10+, CD38+, and/or CD23+[2], with the dual expression of CD5/CD23 being extremely rare and not well documented in the literature without an established prevalence. Here we present the case of a hairy-cell leukemia with concurrent expression of CD5/CD23, highlighting the diagnostic challenge associated with this unusual phenotype, along with a brief review of the literature.

## Case presentation

**Figure 1:**
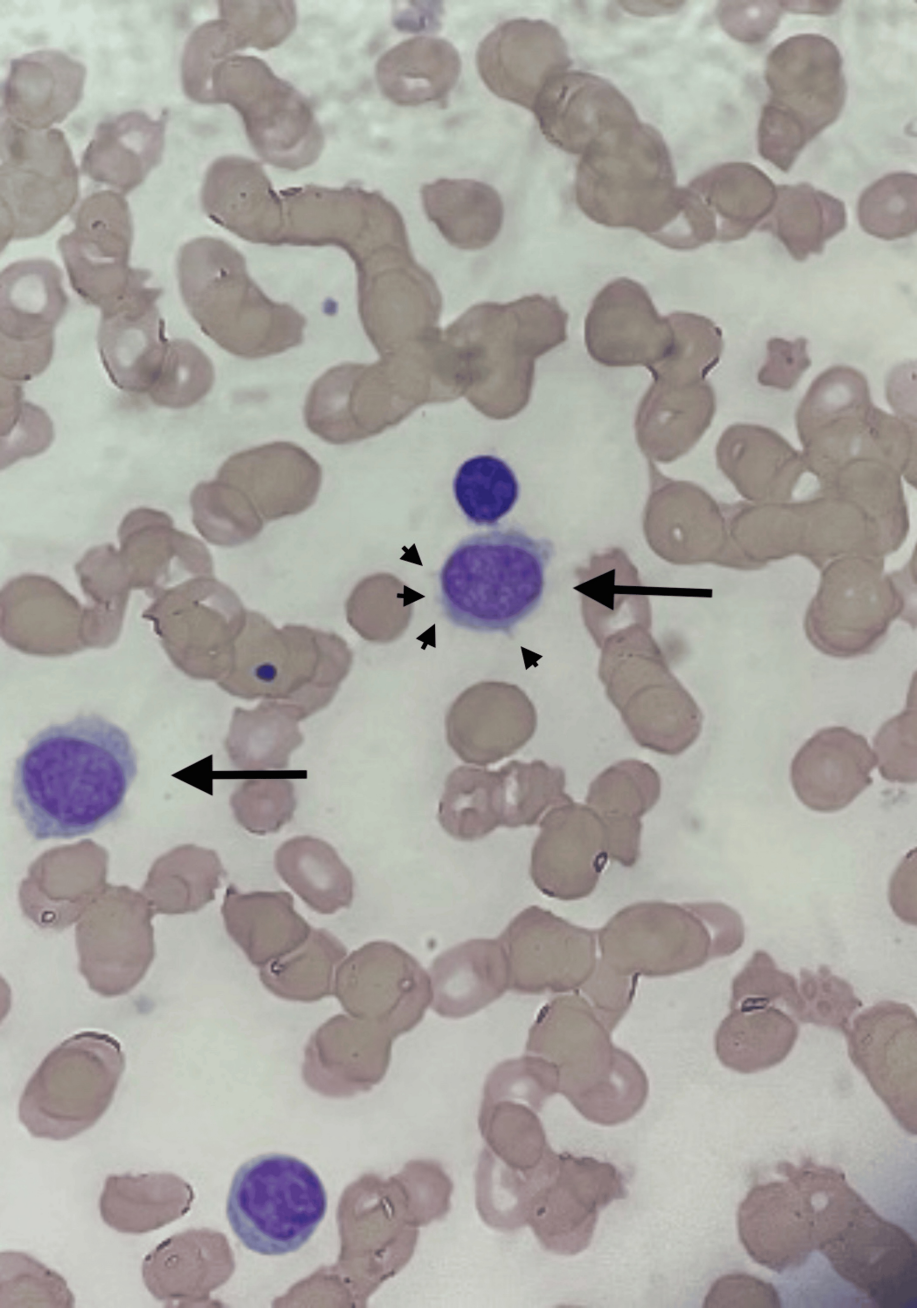
Peripheral smear revealing hairy cells (black arrows) along with their cytoplasmic projections (arrowheads)

A 78-year-old man presents for chest pain and shortness of breath of 2 days duration associated with poor appetite and a 50 lbs weight loss over 6 months. His past medical and surgical history includes gastric adenocarcinoma treated with a partial gastrectomy 20 years ago and a splenectomy. Family history included a father with prostate cancer. He denied occupational exposure. The physical exam was unremarkable. A complete blood count showed a white blood cell count of 10.32 × 103/µL (neutrophils 13.8%, lymphocytes 74%), a hemoglobin of 6.4 g/dL, and a platelet count of 62 × 103/µL. Peripheral smear showed an increase in atypical lymphocytes that were small to intermediate in size, with oval to round nuclei, bland ground-glass, somewhat open chromatin, abundant and pale blue cytoplasm, and spindle-shaped (Figure 1).

**Figure 2:**
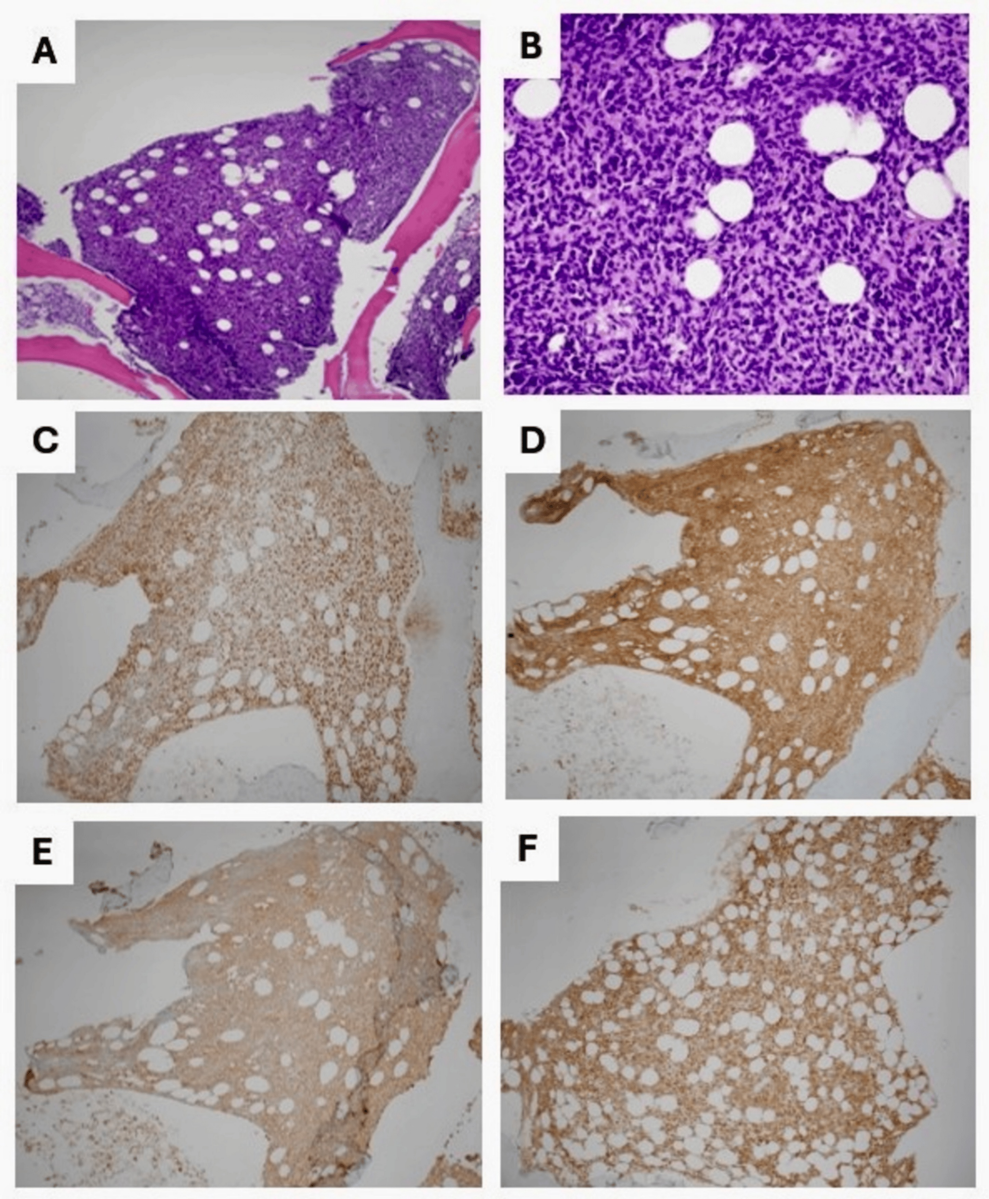
Bone marrow core biopsy and Immunohistochemical Findings. (A) H&E stain at 100X magnification of bone marrow core biopsy showing marked lymphoid infiltrate. (B) H&E at 400X magnification demonstrates that the lymphoid infiltrate is mostly composed of small to intermediate-sized atypical lymphocytes. At the same magnification level (400x), these neoplastic cells are strongly and diffusely positive for PAX5 (C), CD25 (D), and Annexin 1 (E) with aberrant expression of CD5 (F). CD: Cluster of differentiation, H&E: Hematoxylin and eosin, PAX5: Paired box protein 5.

Suspicion for leukemia was raised in the setting of anemia, thrombocytopenia, abnormal peripheral smear, and significant weight loss. Therefore, a bone marrow biopsy was obtained, but unfortunately, the clot and aspirate were noncontributory. Immunohistochemical stains were performed on core biopsy specimen (Figure 2) and showed atypical lymphocytes that are small to intermediate B-cells (PAX5+/CD3-), approximately 80%~90% of total cellularity, positive for CD5, BCL1 (cyclin D1, stained in variable intensity), CD25 (strong and diffuse), annexin 1 (strong and diffuse), TRAP (tartrate-resistant acid phosphatase, stained in variable intensity), CD23 (rare and scattered), while negative for BCL6, CD10, CD123 and SOX11.

A peripheral blood flow cytometry detected a clonal B cell population consistent with involvement by a B-cell lymphoproliferative neoplasm expressing the following phenotype: positive for kappa, CD5, CD23, CD20 (bright), CD200, CD11C, CD25, CD123, and CD103, and negative for CD38 and CD81. Due to the aberrant expression of CD5, a fluorescence in situ hybridization (FISH) test was performed, which was negative for an underlying *CCND1-IGH *gene (cyclin D1-immunoglobulin heavy chain) rearrangement, excluding an underlying mantle cell lymphoma. Additionally, a next-generation sequencing (NGS) test revealed the presence of a *BRAFV600E* mutation, confirming the diagnosis of HCL (Table 1). The patient received one cycle of cladribine, with normalization of his blood counts 4 months later.

**Table 1:** Summary of flow cytometry, cytogenetic, and molecular findings *BRAFV600E*: B-Raf proto-oncogene, Valine 600 glutamic acid, *CCND1-IgH*: Cyclin D1-Immunoglobulin heavy chain, FISH: Fluorescence in situ hybridization, NGS: Next-generation sequencing

Category	Test/Marker	Result
Flow Cytometry	Kappa	Positive
	CD5	Positive
	CD20	Positive
	CD23	Positive
	CD25	Positive
	CD11c	Positive
	CD103	Positive
	CD123	Positive
	CD200	Positive
	CD38	Negative
	CD81	Negative
FISH	*CCND1–IGH* rearrangement	Negative
NGS	*BRAFV600E* mutation	Positive

## Discussion

Brief review of the literature on CD5/CD23-positive HCL 

The concurrent aberrant expression of CD5 and CD23 is exceedingly rare. Two case reports have mentioned this aberrancy. 

Jain et al. reported a case of a CD5-positive classical HCL with a heterogeneous CD23 expression, marking the first case of this dual aberrancy [3]. Though it is important to note that NGS testing was negative for the presence of the *BRAFV600E* mutation, which is considered a hallmark for the diagnosis of classical HCL. Though extremely uncommon, the absence of the *BRAF* mutation has been described in the literature [4].

The second case was reported by Barton and Edmonds of a patient who presented with lymphocytosis and was diagnosed with HCL, supported by the presence of an immunophenotype characteristic of classic HCL on peripheral blood flow cytometry, as he refused to undergo a bone marrow biopsy. Initially CD5+ but CD23-, but CD23 positivity was detected one and a half years later. Cladribine therapy was started shortly after diagnosis due to the worsening of his anemia. A PCR-based assay was employed 10 months after the initiation of treatment, but failed to detect an underlying *BRAFV600E *(Table 2) [5].

**Table 2:** Immunophenotypic and molecular features of reported cases of HCL with CD5 and CD23 co-expression. *BRAFV600E*: B-Raf proto-oncogene, Valine 600 glutamic acid, CD: Cluster of differentiation, FMC-7: Flinders Medical Centre-clone 7, HCL: Hairy cell leukemia, HLA-DR: Human leukocyte antigen-DR isotype, IGHV: Immunoglobulin heavy-variable, N/A= Not available, +: Positive, -: Negative.

Surface antigen	Patient 1	Patient 2	Patient 3
CD5	+	+	+
CD10	−	N/A	−
CD11c	+	+ Bright	+
CD19	+	+	+
CD20	+ Bright	+ Bright	+ Bright
CD22	+	+ Bright	−
CD23	+ Heterogeneous	+ Dim	+
CD25	+	+	+
CD103	+	+	+ Dim
FMC-7	−	+	N/A
HLA-DR	N/A	+	−
Lambda	−	+	−
Kappa	−	N/A	+
Annexin A	+	N/A	+
BRAFV600E	−	−	+
Other	−	Trisomy 12	IGHV 3-23
Reference	[3]	[5]	Current case

It is important to note that a negative result does not definitively exclude a* BRAFV600* mutation, as false-negative results can occur with the cobas® 4800 *BRAFV600* mutation test (Roche Diagnostics, Indianapolis, USA). It has a detection limit of 5% mutant alleles, and the flow cytometry after multiple months of cladribine therapy has identified only a 0.9% population of HCL [5]. Additionally, this assay may miss non-*BRAFV600E* mutations [6].

Diagnostic considerations and differential diagnosis

Typically, the diagnosis of classic HCL is established upon the combination of identification of hairy cells, a distinctive immunophenotypic profile including positivity for CD11c, CD25, CD103, CD123, tartrate-resistant acid phosphatase, and annexin A1 on flow cytometry and/or IHC, and the detection of *BRAFV600E* mutation [4].

Co-expression of both CD23 and CD5 is seldom described in the literature, and when present, it questions the accuracy of the diagnosis. The diagnoses of classical HCL in the case reports of Jain et al. and Barton and Edmonds were supported by the typical morphological appearance and the expression of CD25/CD103 [3,5]. Notably, our case is unique since it is the only reported case demonstrating *BRAFV600E* positivity.

Historically, the HCL variant, first described in 1980**, **was eventually recognized as a distinct entity in the 2008 World Health Organization (WHO), and it typically lacks the expression of CD25, CD123, or CD200 [7]. Due to the uniform absence of *BRAFV600E* mutation and different clinical course experienced by patients with this variant, in the latest *World Health Organization Classification of Hematolymphoid Tumors*, it was replaced by splenic B-cell lymphoma/leukemia with prominent nucleoli [8].

*BRAFV600E* arises from a point mutation in the gene encoding the serine-threonine kinase, where thymine is substituted by adenine in exon 15, and it is present in nearly all cases of classical HCL. It ultimately leads to a downstream activation of the *RAS-RAF-MEK-ERK* signaling pathway, promoting cellular growth and resistance to apoptosis [1,4,9]. Absence of this mutation in classical HCL has been reported, though very infrequently, and mostly in cases where the immunoglobulin heavy-variable (*IGHV*) 4-34 gene was unmutated [1,4].

Hence, in these cases where aberrancy leads to ambiguity, the diagnosis of classical HCL would be defined by the presence of typical clinical, morphological, immunophenotypic, and cytochemical features of classic HCL [4].

When we investigated the two CD markers in question individually, CD5 positivity occurred in less than 10% of HCL cases, and with a fourfold increased frequency of variant HCL in comparison to the classical counterpart in 2 studies [3,4]. A review by Robak et al. suggested that CD5 positivity portended a worse prognosis [4]. On the other hand, they noted that CD23 positivity occurred more frequently, approximately in 20% of HCL patients [4]. Additionally, in a retrospective study by Maitre et al., CD23 positivity was observed more frequently in classic HCL and was associated with a better prognosis [1]. It is unclear what the significance of the dual positivity is.

In the two case reports mentioned in this article, as well as ours, the dual expression of CD23 and CD5 occurred in classical HCL, with the two case reports being* BRAFV600E* negative [3,5].

This dual positivity questions the diagnoses; therefore, additional workup is needed to confirm our final diagnosis. CD23 positivity and absence of t(11;14) translocation on cytogenetics help distinguish HCL from an underlying mantle cell lymphoma (MCL), though few cases have been described where MCL mimics an underlying chronic lymphocytic leukemia (CLL) through expression of CD23 [10]. Furthermore, the presence of typical markers of HCL, such as CD11c, CD103, and CD123, helps distinguish it from CLL (Table 3). In our patient, bone marrow stain was positive for CD5, CD23, and cyclin D1, while FISH confirmed the absence of t(11;14). Although the CD123 stain was negative, its expression was detected by flow cytometry. Such discrepancies may occur due to various factors, including the higher sensitivity of flow cytometry for low-level antigen detection, tissue processing factors in IHC staining, like fixation and decalcification, and differences in reagents used for flow and IHC. These reagents, consisting of anti-CD123 antibodies, can be from different manufacturers or directed against distinct epitopes of CD123, all of which can contribute to platform-dependent variation in antigen detection. 

**Table 3:** Immunophenotypic comparison of the present case with typical hairy cell leukemia, chronic lymphocytic leukemia, and mantle cell lymphoma. *BRAF V600E*: B-Raf proto-oncogene, Valine 600 glutamic acid, *CCND1-IgH*: Cyclin D1-Immunoglobulin heavy chain, CLL: Chronic lymphocytic leukemia, HCL: Hairy cell leukemia, MCL: Mantle cell lymphoma, +: Positive, -: Negative.

Test / Marker	Our Patient	Typical HCL	CLL	MCL
Surface Light Chain	Kappa	Kappa or Lambda	Kappa or Lambda, Dim	Kappa or Lambda
CD5	+	-	+	+
CD20	+, Bright	+, Bright	+, Dim	+, Bright
CD23	+	-	+	-
CD25	+	+	+/-	+/-
CD11c	+	+	-	-
CD103	+	+	-	-
CD123	+	+	-	-
CD200	+	+	+	-
*CCND1–IGH* rearrangement	-	-	-	+
*BRAFV600E* mutation	+	+	-	-

Finally, treatment of classical HCL includes purine analogues with excellent responses [11]. Of the two case reports cited in our manuscript, one achieved a partial response and the other a complete response [3,5]. Our patient achieved normalization of his hematological parameters 4 months after treatment. In summary, it appears that CD5/CD23 positivity doesn’t affect the response to cladribine in all three cases

## Conclusions

Our case report underscores the diagnostic challenge associated with HCL and highlights the importance of a well-structured classification system, particularly when atypical features arise. CD5/CD23 positivity is an unusual presentation with only a limited number of cases documented in the literature. In these rare instances, such aberrant marker expression has been observed in classic HCL. Additional data is needed to determine the significance of this co-expression.
